# Robotic assisted surgery in the United Arab Emirates: healthcare experts’ perceptions

**DOI:** 10.1007/s11701-023-01716-6

**Published:** 2023-09-21

**Authors:** Nasim Barkati, Noura Ntefeh, Ahmad Okasha, Aseel A. Takshe, Rami ElKhatib, Sabrina Chelli

**Affiliations:** Department of Public Health, Canadian University Dubai, Dubai, United Arab Emirates

**Keywords:** Robotic assisted surgery, Perception of care, Medical technology challenges, Surgical innovation, UAE

## Abstract

The adoption of Robotic Assisted Surgery (RAS) has grown around the world. This is also the case in the Middle East and Gulf region and specifically to the United Arab Emirates (UAE). The perception of RAS has been studied in the USA, Europe, and Canada. However, there is limited research on the perception of RAS in the UAE. The study aims to examine the perception of RAS among healthcare experts in the UAE and potential challenges. This qualitative study is based on interviewing healthcare experts in the UAE. Most of the study participants were clinicians and surgeons. In the UAE, RAS is adopted in general surgery, urology, brain surgery, and obstetrics and gynecology. Our findings show that healthcare experts have positive perceptions of RAS. The cost and lack of RAS training program are considered as challenges to adopting RAS in healthcare practices. More research is encouraged to examine perception variations with surgical practices in the UAE, Gulf and the Middle East.

## Introduction

Robotic Assisted Surgery (RSA) can be defined as a procedure using small instruments that are mounted on a structure such as a robotic platform and is controlled by a surgeon operating a computer-controlled interface [1].

The use of RAS has grown significantly around the world [2]. For instance, the main provider of RAS systems, Intuitive Surgical Inc., has shipped 5770 systems around the world [3]. To provide a historical background, Puma 200 was the first surgical robot to be used in clinical practice, and it was introduced in the same year as the first laparoscopic cholecystectomy. The robot was first used for stereotaxic brain biopsy, with the surgeon positioning the robot’s arms to perform the procedure [4]. Robots were also introduced in orthopedics to execute treatments with a defined mathematical and mechanical approach that reduced tissue tactility and tissue fragility.

Robotic surgery advanced with second generation surgical robots such as the Imperial College London’s PROBOT which has soft-tissue surgical capabilities. In 1998, Zeus surgical robot was introduced followed by the Da Vinci system. The Zeus system was used to perform the first beating-heart coronary operation, and in 2001, it was used to perform a surgical operation with the code Lindbergh operation. Like the Lindberg flight across the Atlantic Ocean, it was the first trans-Atlantic operation performed using a tele-robotic system, with a robotic surgical device by surgeons in the USA and a patient in France. The market consolidated with the acquisition of Da Vinci by the parent company of Zeus. Intuitive Surgical. It marketed commercial surgical robots under the name Da Vinci [4].

The purpose of this research is to examine the perception of RAS among healthcare workers in the UAE. RAS is an emerging and a growing field and is expected to have a positive impact on the surgical practices across the world and in the UAE. However, there is a lack of studies on the perception of RAS in the UAE. The next section illustrates literature that examined the use and perception of robotic surgery, followed by methodology, results, discussion, and conclusion.

## Literature review

RAS has gained popularity in fields requiring precision, stability, depth perception and attention to intricate details that can often prove a surgery quite complex [5–8]. From the year 2017 till the beginning of 2018, the use of da Vinci systems had increased by 13% and kept growing annually. Surgical documentations of the year 2010 revealed the application of RAS in the Middle East was more widely used in the following surgical fields, in decreasing order: urology, gynecology, general surgery, pediatrics and cardiac surgery (Azhar et al.). In 2017, it was reported that these documentations increased by 3.4-fold in urology alone [5]. According to Azhar et al. the UAE had possessed 3 da Vinci Surgical Systems amongst the 44 scattered in the Middle East with KSA holding the most at 19, the total representing only 1% worldwide [5]. These numbers are quite impressive when compared to a previous paper published in 2012 that reported only three countries acquiring the robotic systems, KSA holding the most of a total of ten [9]. This number had almost doubled within only a few years. This demonstrates the start of a potentially new acceptance and integration of robot surgical systems in the field of healthcare. Multiple case reports in several fields of surgery using RAS in cases such as extended hysterectomy with pelvic node dissection, pyeloplasty, radical prostatectomy, anorectal malformation repairs and retinal microsurgery to name a few, have demonstrated a promising future, [6, 9–12].

When taking into consideration such radically new technology, it is important to take in the perception of such use by the healthcare professionals. What are the benefits to the healthcare system, and do they outweigh the challenges and disadvantages they may pose? Is it cost-effective? What training is required for proper use of the robotic systems?

The introduction of RAS in surgical practice has come with both numerous advantages and shortcomings. RAS offers many benefits including reducing blood loss, reducing surgical complications, revisions, and post-operative recovery time, better field of vision, removal of hand tremor [8–10, 13, 14]. Additionally, surgeons’ experiences reported facilitation in surgical site access using RAS where it would have otherwise proven to be difficult if done solely laparoscopically [9], thereby increasing accuracy. According to a survey on the perspective of urologists in RAS, 56.8% would recommend RAS to family members over open or laparoscopic cystectomy [10]. Some reports, however, showed a prolonged surgical or set-up time for procedures [7, 9], and/or no additional benefits with the application of RAS in certain procedures such as in pediatric bariatric surgeries resulting in its eventual discontinuation [9] and in general surgery which lacked evidence of improved patient outcome [7]. More recent advances in surgical robotic systems such as the da Vinci Xi Generation were designed to overcome certain limitations faced in previous robotic systems by, for example, improving the facility of the docking maneuver to lessen the total operative time [15]. Furthermore, robotic systems are still being created and assessed for its clinical use in certain fields such as TORS (Transoral Robotic Surgery) which has not yet been fully instated in routine clinical practice due to high cost, low dexterity, increased time, and lack of evidence to demonstrate an overall patient benefit [16]. More evidence is therefore needed to support RAS being more effective than other surgical methods despite its benefits [8, 11, 17]. Nevertheless, its overall popularity in the Middle East was mainly hampered by the low case demands for RAS, too few doctor referrals, and overall demographic factors such as younger population age resulting in less illnesses in need of such procedures relative to other regions in the world [9]. Other challenges healthcare systems may face in the implementation of RAS include the high cost of purchasing a da Vinci robotic system and maintenance expenditure, as well as the associated surgical procedure cost which amounts to $1.5–2.2 million [7, 8, 10].

As with any new technology for such intricate procedures, one would have to take into consideration the training hours required for physicians to be able to use robotic surgical systems for maximal efficiency [17]. This process can become both challenging and time consuming as surgeons adjust to a new way of operating but once mastered, gives obvious benefits such as superior surgical field visualization leaving less room for error [6, 18]. Albeit RAS being more popular and recognized in Western countries [9, 11], surgical centers like the Qatar Robotic Surgery Centre have been established in the Middle East to offer robotic surgical training for healthcare staff involved [6]. In the UAE, several hospitals have implemented RAS as detailed later in this section. Telemedicine is also another option to offer similar training experience by remote experts all around the world [6]. In a study done in Saudi Arabia to evaluate the opinion of urologists on RAS, 72% of those surveyed found that training with RAS should be incorporated into their surgical career path [5]. In a separate study involving Canadian resident urologists, 57% of those surveyed with access of a da Vinci robotic system and 61% without access found the addition of RAS to their training to be valuable [19]. Since the implementation of RAS is still a new and emerging concept, there is still no clear consensus agreed upon worldwide on the qualifications necessary for a surgeon to perform RAS which could raise medicolegal issues [7, 20].

To date public and healthcare experts’ perceptions about robotic surgery in healthcare are largely unknown, yet some minor studies have investigated the topic. The public perception of robotic surgery was studied by Boys et al. The findings showed that 86% of the respondents were aware of RAS and thought of it as laser surgery or a scalrless surgery [21]. The perception of RAS is very important to examine since it can facilitate the adoption of RAS in the surgical field. In addition, its expected that the futuristic medicine will rely heavily on RAS [21].

Currently, there are seven robotic systems in the UAE mainly in Dubai, Abu Dhabi, and Sharjah. Most information on robotic surgical systems pertaining to the UAE were found on healthcare institutional websites, owing to very few publications on RAS in the Middle East. In the UAE, several hospitals have implemented robotic surgery programs, such as Cleveland Clinic Abu Dhabi, American Hospital Dubai, and Al Zahra Hospital Dubai The American Hospital in Dubai was the first healthcare institution to establish a 4th generation da Vinci robotic system and are on their way to implement the ROSA Knee System in orthopedic surgery to provide even higher quality patient care [22]. Additionally, the Clemenceau Medical Center in Dubai have an integrated Robotic Center of Excellence with an 11th Generation DaVinci surgical system [23]. Al Zahra Hospital Dubai also integrated RAS in their delivery of care using the Versius Robotic surgical system [24]. Mediclinic City Hospital implemented the use of the da Vinci XI HD 4 arm Robotic System to their facility [25]. Also, Al Qassimi Hospital for Women and Children in the emirate of Sharjah purchased a da Vinci robotic system. In the Emirate of Abu Dhabi, Cleveland Clinic Abu Dhabi purchased a da Vinci surgical system, offering a great variety of RAS in various surgical fields [26]. Moreover, in early September of 2020, Sheikh Shakhbout Medical City performed its first RAS and marked the launch of their Robotic Surgery Training Program [27].

It is in every physician’s best intention to provide the utmost care as well as latest medical and surgical practices for their patients. Considering patient factors that would result in a preference for a surgery performed by the hands of a skilled surgeon alone as opposed to with robotic assistance remains an important deliberation for the future establishment of RAS [13, 28]. In a study published in 2020 looking at public awareness on RAS in Kuwait, less than half of the participants were unsure of its safety while merely 29.7% felt it was safe and 22.7% did not [29]. Furthermore, only 36.8% out of 1,087 citizens had been aware of RAS at the time [29]. Moreover, in a study conducted in Europe on the trust of the general population on RAS, they established that the perception of RAS was influenced by a myriad of factors that ranged from sociodemographic factors, past experiences with robots, knowledge, expectations, perception of robots with regards to the level of care provided by the experience and training of the surgical team with RAS [13]. Amongst their concluding results, they found that individuals with more knowledge and perceived beneficial expectations of robots had less trust in their surgical use [13]. With this regard, meeting a good standard of care and gaining the public’s trust and awareness of RAS by exploring core issues will be undeniable for the proper implementation and use of RAS now and soon.

Ultimately, RAS has a great potential to upgrade medicine and improve surgeries for both healthcare professionals and patients. However, its application to this day is more widely used in certain surgical specialties and procedures more than others due to lack of evidence pointing towards significant improvement in surgical outcome despite its many benefits [10]. Additionally, there is yet to be a clear training criterion agreed globally to obtain the qualifications necessary to operate with RAS. Robotic surgery is more popular in Western countries than in the Middle East as well as its awareness in the general population [29]. Gaining public trust in RAS remains an important factor for the successful implementation of RAS in the foreseeable future. Finally, there are very few published papers on robotic surgery in the Middle East and most information available in the UAE on robotic surgeries are found on healthcare institution websites, making this paper an important contribution to the future of robotic surgery in the UAE.

## Methods

This study is qualitative research that aims at exploring healthcare experts’ perceptions regarding RAS implementation and barriers that impede its adoption in the UAE. Perceptions and experiences can provide information on various barriers, which are not always portrayed clearly using quantitative methods but can only be retrieved from an in-depth understanding of processes and context [30]. A document analysis of the available information on RAS in the UAE was conducted. Documents were downloaded from official websites of hospitals. More than 100 healthcare experts such as surgeons, medical registrars, chief financial officers in the UAE were approached initially by emails and phone calls to request interview slots. Yet, the response rate was 17%. Literature shows that interviewing is the most used method for data collection in qualitative research The participants were interviewed one-to-one using Zoom. Seventeen (17) in-depth interviews with professionals in the field of healthcare in the UAE were conducted between January and March 2022 online. The interviews lasted between 40 and 50 min. Initially, the study’s aim and procedure were clearly explained to the participants and participants’ confidentiality was ensured before obtaining their oral consent. Five open-ended questions were used to guide the interviews. The questions were related to participants’ perceptions of the importance of RAS in the workplace, fields that needed RAS implementation, obstacles in adopting and implementing RAS and the effect of COVID-19 on RAS implementation. All interviews were audio-recorded with the consent of the interviewees. We reached saturation after the seventeenth interview as no new themes were emerging. Guest et al. [31] refers to saturation as having become ‘the gold standard by which purposive sample sizes are determined in health science research.’ A sample of 17 responses can be assumed as valid [32, 33]. The researchers used thematic analysis to code the transcripts. Around 15 initial codes were identified. Similar codes were then grouped into emerging themes that illustrate the barriers to the adopting and implementing RAS in UAE. To avoid bias and ensure the trustworthiness of the study, we aimed to meet the following criteria for qualitative studies [30, 34]. Both, co-authors checking, and peer debriefing was done to ensure the credibility of the research findings. Transferability and consistency of finding was verified to guarantee dependability through describing the research process thoroughly. Participants were encouraged to verbalize their perceptions to ensure confirmability.

## Results

The data has been divided into quantitative and qualitative sections. The quantitative data is provided below in Table 1 and interpretated qualitatively later on throughout this section.

**Table 1:** Quantitative results of the RAS perceptions among healthcare experts in the UAE

	Number	Percentage %
**Participants’ specialty**		
Clinicians	11	65
Non-clinicians	6	35
**Perception of RAS**		
Favorable	15	88
Neutral	2	12
Against	0	0
Improve hospital reputation	16	94
**Medical specialties adopting RAS**		
General surgery	3	18
Urology	3	18
Brain surgery	3	18
Other specialties	3	18
Obstetrics and gynecology	1	6
**RAS adoption challenges**		
Cost and financials	10	59
Technological challenges	1	6
No insurance coverage	1	6
Increase in workload due to RAS	3	18
**Impact of COVID-19 on RAS**		
No change regarding RAS adoption plans	8	47
Change of plans regarding RAS adoption plans	9	53

### Participants role

The participant roles cover clinical and non-clinical duties in healthcare. For instance, 11 of our respondents were clinicians such as orthopaedic surgeons, urologists, laparoscopic surgeons, bariatric surgeons, and pharmacists. Six of the of respondents were non-clinicians such as CEOs, healthcare manager, health informatic specialists, and a program manager.

### Perception of RAS

Regarding the perception of robotic surgery in general, 52.9% of the study respondents perceive robotic surgery as a very useful medical technology and are planning to use it. As shown in Table 1, around 35% 0.3 percent of the respondents agree that robotic surgery is a useful technology. However, their current healthcare organizations are not ready to use it. Since most of the respondents are clinicians, they are planning to use it within their practices. About less than 6% of the study participants believed that robotic surgery is expensive and complex. Additional 6% are against the use of robotic surgery in their practices or their organizations.

In terms of provision of robotic surgery, 29.4% of the survey respondents already offer robotic surgery to their patients, and 35.3% of the respondents are not planning to offer the service. The rest of the respondents maintained that there is a plan to offer the service in the future, in which 11.8% are planning to offer it in two years, 17.6% in three years, and 5.9% in 1 year.

Concerning the reason for implementing robotic surgery or planning to implement it, 23.5% of the survey respondents did not have an answer due to the fact that they do not offer robotic surgery services, 47.1% of respondents indicated that their hospitals decided on offering the service, for better clinical quality outcomes. Hospital’s reputation was the answer of 23.5% of the respondents, and the last 5.9% of our respondents implemented robotic surgery services to increase hospital’s revenues.

With respect to whether offering robotic surgery services can enhance the performance and reputation of the hospital, 94.1% of the participants indicated that RAS could indeed enhance the reputation and performances of the hospital.

### Medical specialities adopting RAS

**Fig. 1:**
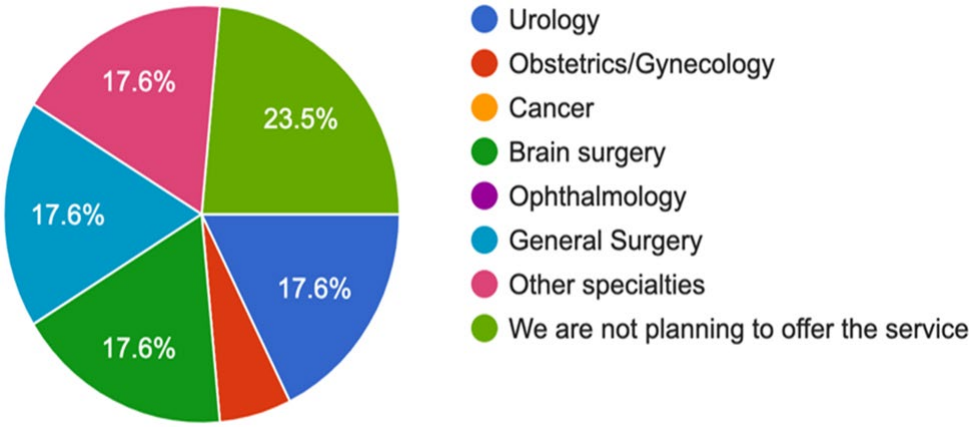
RAS adoption based on medical specialties

Figure 1 shows that RAS is planned to be used in general surgery, urology, and brain surgery specialties. Around 5.9% of the study participants are planning to use the service for obstetrics and gynaecology, and 17.6% of the respondents indicated that they are planning to use RAS in other specialities than general surgery, urology, brain surgery, and obstetrics and gynaecology. Figure 1 also shows that 23.5% of the study respondents indicated that they are not planning to offer RAS.

### RAS adoption challenges

**Fig. 2:**
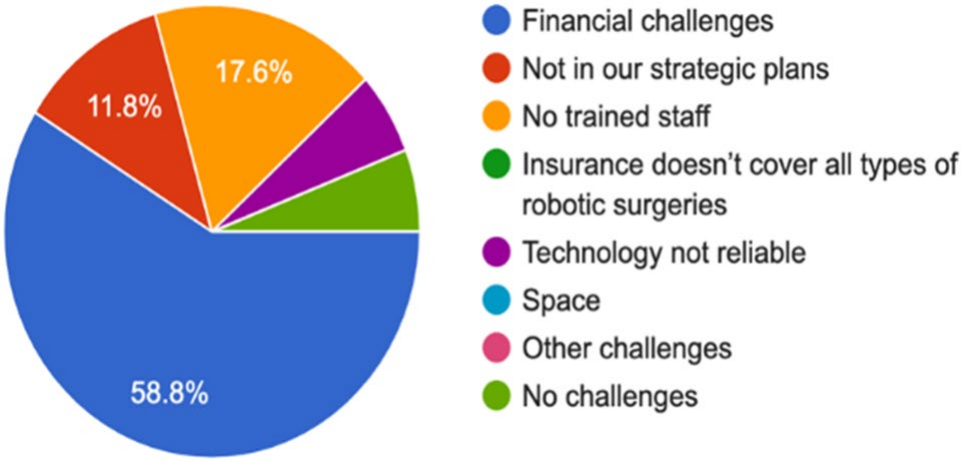
Study participants’ perceived challenges of adopting RAS

In terms of the challenges that might prevent the implementation of robotic surgery, 58.8% of the study respondents highlighted financial challenges as shown in Fig. 2. According to the participants, RAS is a costly service to implement in their practices. Around 17.6% of the study respondents found that one of the obstacles they faced was that there was no trained staff on RAS technologies, 11.8% mentioned that it is not in their strategic plans, 5.9% believe that the technology is not reliable, and the last 5.9% mentioned that they did not face any challenges while implementing the service.

With respect to the fact that whether the workload will increase or decrease when RAS will be implemented, about 52.9% of the participants believe that there is no change in the medical staff workload, and 29.4% of the respondents think that the workload will be reduced. Only 17.6% of the study respondents believe that the workload will increase.

When comparing traditional surgeries to robotic surgeries, 18% respondents mentioned that traditional surgeries are cost effective. Around 44% respondents to this survey indicated that they have more flexibility with traditional surgery.

### Impact of the COVID-19 pandemic on RAS

Concerning the impact of the COVID-19 pandemic on the hospital plan to provide robotic surgery, 47% of the respondents indicated that COVID-19 pandemic did not change their plans and 53% of respondents indicated that COVID-19 changed their plans with regard to implementing robotic surgeries.

## Discussion

The following section analyses and interprets that data from the interviews. Moreover, it explains the significance and implications of the information.

### Study participants

Most of the study respondents were surgeons and clinicians and thus are better able to provide more relevant perception of the utility of robotic surgeries in their specialties. Our findings are similar to previous studies on the perception of the use of RAS in Saudi Arabia [10] and Canada [19]. Other studies on RAS examined public opinions in Kuwait [29], (Buabbas et al.) and globally [13, 35, 36]. Interestingly, one study found a gender difference with regard to the perception of robotic surgery. The study found females respondents tend to dehumanize RAS while males tend to humanize the RAS [28].

### Perception of RAS

Most of the study participants have a positive perception of RAS. Other studies examined RAS perception in Europe, US, Canada, Kuwait, and Saudi Arabia and in general found a positive awareness of RAS and a positive perception of RAS in surgery [5, 13, 19]. For instance, a US study on RAS found that nearly half of the participants have trust in the utility of RAS. Even more interesting is that when it comes to cancer diagnosis, respondents are more likely to trust RAS than physicians [36].

The study results indicated that a third of the study participants are already using RAS. This is similar to previous studies [5, 29, 37]. Third of the respondents indicated that their infrastructures are not ready. Since most of the respondents are clinicians, they are planning to use it within their practices. Around 35% of the respondents indicated that their organizations are not planning to offer RAS. This could be due to limitations of space needed and large initial capital investment required [38].

Approximately, 47.1% of study respondents clarified that their hospitals decided on offering the service to improve clinical outcomes [6, 10, 13, 18]. With regard to the reason for implementing robotic surgery or planning to implement it, 23.5% of the survey respondents did not have an answer due to the fact that they do not offer robotic surgery services. Around 23.5% of the respondents indicated that hospitals or their practices are offering RAS to improve the reputation of their hospitals. A large percentage of the hospitals in the UAE are privately owned hospitals, therefore, adopting the latest medical technologies is considered as an important marketing tool to enhance their reputations. Lastly, around 5.9% of our respondents implemented robotic surgery services to increase hospital’s revenues.

### Medical specialities adoption RAS

With the advances in medical technology, more and more medical specialities are adopting RAS. Similar to the findings of other studies, our findings show that 52.8% of the study respondents indicated that RAS is being used in general surgery [39] urology and brain surgery in equal proportion [2, 19, 40]. Each of these specialities had a 17.6% of the study respondents. Around 5.9% of the study participants are planning to use the service for obstetrics and gynaecology [40]. About 17.6% of the study respondents indicated that they are planning to use RAS in specialities other than general surgery, urology, brain surgery, and obstetrics and gynaecology [6]. The study-maintained RAS will be a mainstream technology in all surgical specialities.

### RAS adoption challenges

Robotic surgery is an expensive technology if we compare it with traditional medical methods. For instance, the cost of a spine surgery RAS system can range from $500,000 to $1 million US Dollars. There are also annual operational and maintenance costs [41]. It is estimated that the medical technology cost alone of each surgical procedure using robotic surgery system is $1866 US Dollars [42]. The high cost was also reported in our study as the main challenge of implementing RAS in the UAE [39]. In addition, the second main challenge mentioned by the participants is the lack of technical training of surgeons [10, 19, 20]. Other studies indicated that other challenges are related to insurance coverage [40] and issues related to communication [14].

As indicated earlier, many physicians perceive RAS to improve the patient outcome and improve precision [6]. Most of the study respondents did not perceive robotic surgery as adding to their workload. On the contrary, a third of the respondents believe it will decrease their workload [19].

When it comes to the differences between RAS and traditional surgeries, similar to other studies, the study respondents reported flexibly as the main advantage of traditional surgeries over robotic surgeries [10, 13, 18].

### Impact of COVID-19 pandemic on RAS

COVID-19 pandemic did impact hospital plans to implement robotic surgery since most of the hospitals were focusing on safety and how to go back to normal operations [43]. In addition, many elective surgeries during the COVID-19 pandemic were delayed or cancelled. RAS were mostly used in elective surgeries in the UAE according to our respondents. Therefore, the number of RAS declined during COVID-19 pandemic. Elective surgeries were no longer being done in the public sector. As the UAE and the whole world is recovering from the impact of the COVID-19 pandemic, we expect the usage of RAS to increase in the UAE.

## Conclusion

The purpose of this paper is to study the perception of healthcare experts on the use of RAS in UAE. Medical technology is advancing at a significant speed. RAS is one of the available medical technologies which has a great potential improve the outcomes of care. With better precision, shorter surgical time, and smaller incisions, it is expected that patients will heal better and develop less surgical complications.

Our findings show that most of the study participants have a positive view on RAS, indicating that it is correlated with improving medical outcome and reducing surgical complications. The application of RAS within medical specialities is still limited to urology, general surgeries, and brain surgeries surgical procedures. Improving hospital reputation was cited as a main reason to adopt RAS. Perceived challenges included the high cost of RAS services and the lack of technical training. RAS device manufacturers could develop better creative strategies to lower the costs of ownerships to healthcare providers. The study respondents perceive that the COVID-19 pandemic lowered the adoption of RAS in the UAE. As the UAE is recovering and removing the restrictions following the COID-19 pandemic, it is expected that more providers will use RAS.

One of the limitations of this study is the relatively limited sample size. A larger sample size may reveal variations in the perception and adoption of RAS among clinicians.

More research should measure public opinions of RAS in the UAE. In addition, more research in the UAE is needed for measuring the outcomes of RAS and potential side effects.

## Data Availability

The datasets generated during and/or analyzed during the current study are available from the corresponding author on reasonable request.
